# Incident colorectal cancer screening and associated healthcare resource utilization and Medicare cost among Medicare beneficiaries aged 66–75 years in 2016–2018

**DOI:** 10.1186/s12913-022-08617-8

**Published:** 2022-10-03

**Authors:** Suying Li, Lesley-Ann Miller-Wilson, Haifeng Guo, Madison Hoover, Deborah A. Fisher

**Affiliations:** 1 Chronic Disease Research Group, Hennepin Healthcare Research Institute, 701 Park Avenue, Suite S2.100, Minneapolis, MN 55415 USA; 2 Exact Sciences Corporation, Madison, WI USA; 3 Department of Medicine and Duke Clinical Research Institute, Duke University, Durham, NC USA

**Keywords:** Colorectal cancer, Colorectal cancer screening, Healthcare costs, Healthcare utilization, Incidence, Medicare

## Abstract

**Background:**

While prevalence of up-to-date screening status is the usual reported statistic, annual screening incidence may better reflect current clinical practices and is more actionable. Our main purpose was to examine incident colorectal cancer (CRC) screening rates in Medicare beneficiaries and to explore characteristics associated with CRC screening.

**Methods:**

Using 20% Medicare random sample data, the study population included 2016–2018 Medicare fee-for-service beneficiaries covered by Parts A and B aged 66–75 years at average CRC risk. For each study year, we excluded individuals who had a Medicare claim for a colonoscopy within 9 years, flexible sigmoidoscopy within 4 years, and multitarget stool DNA test (mt-sDNA) within 2 years prior; therefore, any observed screening during study year was considered an “incident screening”. Incident screening rates were calculated as number of incident screenings per 1000 Medicare beneficiaries. Overall rates were normalized to 2018 Medicare population distributions of age, sex, and race.

**Results:**

Each year, > 1.4 million individuals met the inclusion/exclusion criteria from > 6.5 million Medicare beneficiaries. The overall adjusted incident CRC screening rate per 1000 Medicare beneficiaries increased from 85.2 in 2016 to 94.3 in 2018. Incident screening rates decreased 11.4% (22.9 to 20.3) for colonoscopy and 2.4% (58.3 to 56.9) for fecal immunochemical test/guaiac-based fecal occult blood test; they increased 201.5% (6.5 to 19.6) for mt-sDNA. The 2018 unadjusted rate was 76.0 for men and 110.4 for women. By race/ethnicity, the highest 2018 rate was for Asian individuals and the lowest rate was for Black individuals (113.4 and 72.8, respectively).

**Conclusions:**

The 2016–2018 observed incident CRC screening rate in average-risk Medicare beneficiaries, while increasing, was still low. Our findings suggest more work is needed to improve CRC screening overall and, especially, among male and Black Medicare beneficiaries.

**Supplementary Information:**

The online version contains supplementary material available at 10.1186/s12913-022-08617-8.

## Background

Colorectal cancer (CRC) is the second leading cause of cancer-related deaths in men and women combined in the United States [1]. Regular CRC screening is important for reducing CRC-related death and CRC incidence, which affects healthcare resource utilization (HCRU) [2]. The US Preventive Services Task Force (USPSTF) previously recommended CRC screening in all adults aged 50–75 years and lowered the age of screening initiation in 2021 to 45 years [3].

Medicare is the federal health insurance program, mainly for people aged 65 years and older. In 2019, Medicare covered about 53 million beneficiaries aged 65 years and older [4]. Medicare coverage for CRC screening has evolved over time; average-risk colonoscopy screening was added in 2001 [5], cost-sharing for preventive services including CRC screening was eliminated in 2011 [6], and multitarget stool DNA test (mt-sDNA) reimbursement was added in 2014 [7].

Reported CRC screening rates vary by data source, study population, and definition. While cumulative rates of up-to-date screening status are a common metric, incident screening rates offer potential advantage of better reflecting current practices and being more actionable for quality improvement efforts. In this study, we had three objectives: 1) to estimate 2016–2018 incident CRC screening rates in US Medicare beneficiaries aged 66–75 years at average risk for CRC, 2) to explore subject characteristics and clinical factors associated with incident screening, and 3) to estimate 2018 HCRU and Medicare spending in individuals who underwent different screening types.

## Methods

### Data source and study population

We used the 2007–2018 20% Medicare random sample data, including enrollment information, demographic characteristics, and medical claims from Parts A, B, and D. The study population included Medicare beneficiaries aged 66–75 years at average risk for CRC and covered under the Medicare fee-for-service (FFS) Parts A and B. We used the USPSTF recommendations [3], as well as published studies in the Medicare population, to define our average-risk cohort [8–13]. In this study, we defined average risk as no personal history of CRC, polyps, or inflammatory bowel disease (ulcerative colitis or Crohn disease) and no confirmed or suspected hereditary CRC syndrome, such as familial adenomatous polyposis or Lynch syndrome (hereditary nonpolyposis colon cancer).

### Study design and study sample

We defined study cohort for each year, 2016–2018. The 2016 study sample included average-risk Medicare FFS beneficiaries who 1) were aged 66–75 years on January 1, 2016; 2) were continuously enrolled in Medicare Parts A and B in 2015 and 2016; 3) had no diagnosis in Medicare claims for history of CRC, polyps, inflammatory bowel disease, or hereditary CRC syndrome in 2015; 4) were not admitted to hospice in 2016; 5) had no Medicare claim for a colonoscopy within 9 years during 2007–2015, flexible sigmoidoscopy within 4 years during 2012–2015, or mt-sDNA within 2 years during 2014–2015; and 6) had no Medicare claim for CRC test on or after a high-risk CRC event during 2016, if such an event was observed during 2016. Claims were available only for years that the beneficiary was enrolled in the Medicare program. The baseline year of 2015 was used to define comorbidities and high-risk events for exclusions. The *Current Procedural Terminology (CPT)*, Healthcare Common Procedure Coding System (HCPCS), and *International Classification of Diseases, Ninth Revision, Clinical Modification (ICD-9-CM)* and *ICD-10-CM* diagnosis or procedure codes for high-risk events are listed in Additional file 1: Supplementary Appendix 1. Follow-up was from January 1 to December 31, 2016, and censored at the date of death, end of Medicare Parts A and B coverage, or development of a CRC high-risk event. Observed incident CRC screening, HCRU, and Medicare costs were determined in the follow-up period. A similar design was used for the 2017 and 2018 samples.

### Defining variables and outcomes

#### Incident CRC screening and annual screening rate

The CRC screenings were identified by *CPT* and HCPCS codes (Additional file 1: Supplementary Appendix 2) from Medicare Part B claims and Medicare Part A outpatient claims. The CRC screening types included colonoscopy, fecal immunochemical test or guaiac-based fecal occult blood test (FIT/gFOBT), and mt-sDNA. As described above regarding the 2016 example, we excluded individuals who had a Medicare claim for a colonoscopy within 9 years, flexible sigmoidoscopy within 4 years, and mt-sDNA within 2 years prior to 2016; therefore, any observed screening from Medicare claim during 2016 was considered an “incident screening”. Incident CRC screening rate in 2016 was calculated as the number of incident screenings divided by the total selected study sample in 2016 and expressed as per 1000 Medicare beneficiaries. Similarly, we calculated the incident screening rate for 2017 and 2018. In addition, we used the direct method to estimate overall yearly screening rates. Overall incident screening rates for 2016 and 2017 were normalized to 2018 Medicare population distributions of age, sex, and race.

#### Baseline demographic characteristics and comorbid conditions

Patient baseline characteristics included age on January 1, sex, race/ethnicity (White, Black, Asian, Hispanic, and other), region (Northeast, Midwest, South, West), and Medicare/Medicaid dual-eligibility status during baseline (yes/no). Patient baseline clinical conditions included a continuous variable for the Charlson Comorbidity Index [14] and presence of comorbid conditions at baseline (yes/no). Patient baseline comorbid conditions were defined by at least one inpatient hospital, skilled nursing facility, home health, or hospice claim; or two hospital outpatient visits, Part B physician visits, or durable medical equipment claims at least 30 days apart within a year. The *ICD-9-CM* and *ICD-10-CM* diagnosis codes for defining the selected comorbid conditions are listed in Additional file 1: Supplementary Appendix 3. These comorbid conditions included arteriosclerotic heart disease, heart failure, cerebrovascular accident/transient ischemic attack, peripheral vascular disease, other cardiac disease, diabetes, anemia, hypertension, hyperlipidemia, chronic kidney disease (excluding dialysis), cancer (excluding nonmelanoma skin cancer), liver disease, chronic obstructive pulmonary disease, and gastrointestinal bleeding.

#### Annual HCRU and Medicare costs for 2018 study sample

For the 2018 sample, HCRU was identified for all-cause hospitalizations, outpatient visits, emergency department (ED) visits/observational stays, and physician visits. Measures of hospitalization included number of all-cause admissions per person per year (PPPY) and total hospital length of stay in days PPPY for the entire study sample and hospitalized patients, respectively. Outpatient visits, ED visits/observational stays, and physician visits were measured as total number of visits PPPY.

In Medicare claim data, the variable of Medicare allowable amount included Medicare paid amounts and patient paid amounts. In our study, Medicare costs were determined by the Medicare paid amount. For the primary analysis, we used Parts A and B claims data to calculate total Medicare costs, including Part A inpatient and outpatient costs (including outpatient ED costs), Part A other costs (skilled nursing facility, home health agency, and hospice), and Part B costs for physician visits and durable medical equipment. In the sensitivity analysis we also included Part D costs (for beneficiaries who had Part D) for prescription drugs in the Medicare total spending. Medicare costs were calculated as $PPPY for individuals who underwent the following screenings: colonoscopy, FIT/gFOBT, and mt-sDNA.

There were small numbers of patients who received a screen from each of the categories in their screening year. To avoid any biased results, we excluded those patients from analysis when examining Medicare costs and HCRU.

### Statistical methods

For patient baseline characteristics, we reported frequency and percentage for categorical variables; χ^2^ test was used for comparing differences between individuals screened and unscreened for CRC. Incident screening rates and 95% confidence interval (CI) were reported as numbers of screened per 1000 Medicare beneficiaries at average risk. Adjusted odds ratio (OR) and 95% CI of incident screening were estimated using generalized estimating equation logistic regression adjusted by demographic characteristics, cohort year, and comorbid conditions. Mean, standard deviation, median, and interquartile range were reported for HCRU and Medicare costs PPPY.

## Results

### Cohort selection and baseline characteristics

In 2016–2018, each year approximately 1.4 million individuals at average risk for CRC met the inclusion and exclusion criteria from the more than 6.5 million Medicare FFS beneficiaries in the 20% random sample. Sample selection with inclusions and exclusions are shown in Additional file 1: Supplementary Table 1. We have compared demographic characteristics between the final selected sample and the general 20% sample before selection (ie, those who lived on January 1, 2018, without requirement of Medicare Parts A and B coverage) for the 2018 study year. Compared to the general 20% sample before selection, the final selected sample had slightly lower mean age (70.1 vs 69.6 years), a lower proportion of females (54.3% vs 53.2%), a higher proportion of White (79.7% vs 82.6%), and lower proportions of Black (9.4% vs 7.8%) and Hispanic (2.5% vs 1.9%).

Table 1 presents demographic characteristics and comorbid conditions by screening status for the 2016–2018 study samples. Distributions of demographic characteristics and prevalence of comorbid conditions by screening status were similar across study years. For each study sample, the age distribution was similar between screened and unscreened individuals; there was a greater percentage of women, a lower percentage with Black race, and a lower percentage with Medicare/Medicaid dual eligibility in screened individuals compared with unscreened individuals. Compared with unscreened individuals, screened individuals had a higher prevalence of the following conditions: diabetes, other cardiac, anemia, hypertension, hyperlipidemia, chronic kidney disease, and cancer.

**Table 1:** Demographic characteristics and comorbid conditions by screening status for Medicare beneficiaries aged 66–75 years, 2016–2018

	**2016**			**2017**			**2018**		
	**Total**	**Screened (121,184)**	**Not screened (1,303,269)**	**Total**	**Screened (133,972)**	**Not Screened (1,334,205)**	**Total**	**Screened (139,319)**	**Not Screened (1,338,319)**
	N	**Column %**		N	**Column %**		N	**Column %**	
Overall	1,424,453	100	100	1,468,177	100	100	1,477,638	100	100
Age (in years)									
Mean (SD)		69.4 (2.7)	69.5 (2.8)		69.5 (2.7)	69.6 (2.8)		69.6 (2.7)	69.6 (2.8)
Median (IQR)		69 (67,72)	69 (67,72)		69 (67,72)	69 (67,72)		69 (67,72)	69 (67,72)
Sex									
Male	670,184	38.09	47.88	688,896	37.94	47.82	691,810	37.76	47.76
Female	754,269	61.91	52.12	779,281	62.06	52.18	785,828	62.24	52.24
Race/ethnicity									
White	1,191,824	84.70	83.57	1,218,095	84.01	82.86	1,220,334	83.57	82.48
Black	112,627	6.27	8.06	116,326	6.30	8.09	115,678	6.04	8.01
Asian	27,515	2.34	1.89	30,621	2.47	2.05	32,142	2.62	2.13
Hispanic	25,023	1.78	1.75	27,506	1.89	1.87	28,611	2.09	1.92
Other	67,464	4.91	4.72	75,629	5.33	5.13	80,873	5.68	5.45
Regions									
Northeast	243,627	17.32	17.08	246,997	16.74	16.83	249,149	16.20	16.93
Midwest	311,138	20.07	22.01	327,524	21.20	22.42	326,351	21.02	22.20
South	566,202	40.50	39.68	574,470	39.74	39.07	572,050	39.05	38.68
West	295,876	21.71	20.68	311,583	22.00	21.14	322,419	23.36	21.66
Missing	7610	0.41	0.55	7603	0.33	0.54	7669	0.37	0.53
Payer Type									
Medicare only	1,273,781	91.19	89.26	1,308,336	90.47	88.98	1,317,655	90.35	89.05
Dual Medicare/Medicaid	150,672	8.81	10.74	159,841	9.53	11.02	159,983	9.65	10.95
Charlson Comorbidity Index									
Mean (SD)		0.53 (1.15)	0.55 (1.25)		0.58 (1.20)	0.60 (1.31)		0.60 (1.23)	0.61 (1.33)
Median (IQR)		0.0 (0.0,1.0)	0.0 (0.0,1.0)		0.0 (0.0,1.0)	0.0 (0.0,1.0)		0.0 (0.0,1.0)	0.0 (0.0,1.0)
Comorbid Conditions									
DM	278,575	20.88	19.43	291,053	21.76	19.63	288,744	21.56	19.33
ASHD	144,003	9.79	10.14	155,749	10.65*	10.60*	154,854	10.46*	10.48*
Heart failure	59,531	3.35	4.26	64,749	3.66	4.49	66,316	3.82	4.56
CVA/TIA	52,344	3.40	3.70	56,171	3.63	3.85	57,078	3.71	3.88
PVD	76,274	4.97	5.39	88,205	5.92*	6.02*	90,298	6.14*	6.11*
Cardiac other	102,547	7.98	7.13	190,352	14.10	12.85	191,734	13.97	12.87
Anemia	86,052	6.68	5.98	94,268	7.10	6.35	94,566	7.03	6.33
Hypertension	575,945	47.39	39.79	625,737	50.57	41.82	625,486	49.91	41.54
Hyperlipidemia	473,287	42.87	32.33	514,025	45.26	33.98	515,440	44.83	33.85
CKD	105,824	7.49*	7.42*	168,084	12.17	11.38	172,787	12.52	11.61
Cancer ^a^	84,375	6.79	5.84	84,468	6.43	5.69	85,362	6.41	5.71
Liver disease	11,106	0.85	0.77	16,708	1.32	1.12	18,710	1.42	1.25
COPD	126,270	8.44	8.90	135,543	9.04	9.25	136,318	9.18*	9.23*
GI bleeding	4744	0.33*	0.33*	5585	0.33	0.39	5531	0.34*	0.38*

*ASHD* Arteriosclerotic heart disease, *CKD* Chronic kidney disease, *COPD* Chronic obstructive pulmonary disorder, *CVA/TIA* Cerebrovascular accident/transient ischemic attack, *DM* Diabetes mellitus, *GI* Gastrointestinal, *IQR* Interquartile range, *PVD* Peripheral vascular disease, *SD* Standard deviation

^a^ Cancer excluding nonmelanoma skin cancer

^*^ *P*-values > 0.05

### Yearly observed incident CRC screening rates and factors associated with incident screening

**Fig. 1:**
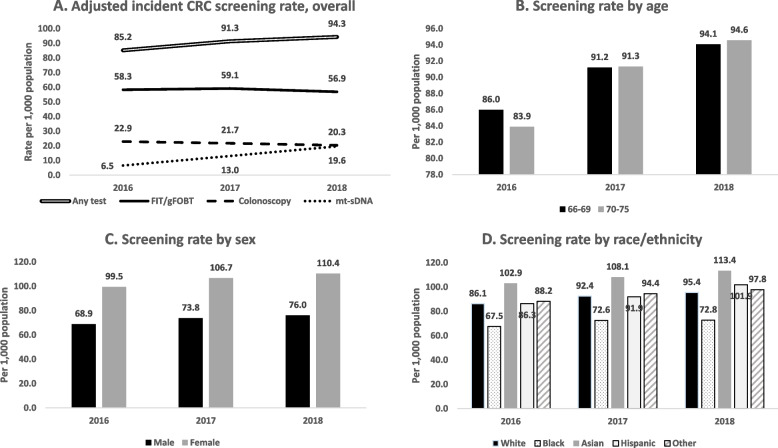
Incident CRC screening rate in 20% Medicare beneficiaries aged 66–75 years at average risk, overall and by demographics. CRC, colorectal cancer; FIT/gFOBT, fecal immunochemical test or guaiac-based fecal occult blood test; mt-sDNA, multitarget stool DNA test

As shown in Fig. 1, the overall age-, sex-, and race-adjusted incident CRC screening rate per 1000 Medicare average-risk beneficiaries increased from 85.2 in 2016 to 91.3 in 2017 and 94.3 in 2018. By screening type, adjusted incident screening rates from 2016 to 2018 decreased 11.4% (22.9 to 20.3) for colonoscopy, decreased 2.4% (58.3 to 56.9) for FIT/gFOBT, and increased 201.5% (6.5 to 19.6) for mt-sDNA.

The 2018 unadjusted overall rate for women was higher than the rate for men (110.4 and 76.0, respectively). By race/ethnicity, in 2018, the highest rate was for Asian individuals and the lowest rate was for Black individuals (113.4 and 72.8). The unadjusted incident screening rates by demographic characteristics for 2018—for any screening and for each screening type—are presented in Table 2. There were race/ethnicity disparities by screening types. For example, the incident screening rates of colonoscopy were 20.7 for White, 18.3 for Black, 15.4 for Asian, and 14.5 for Hispanic individuals; the rates of FIT/gFOBT were 55.9 for White, 46.7 for Black, 90.5 for Asian, and 84.9 for Hispanic individuals; and the rates of mt-sDNA were 21.4 for White, 9.4 for Black, 9.3 for Asian, and 4.1 for Hispanic individuals.

**Table 2:** Incident CRC screening rates by screening types in 2018 Medicare beneficiaries aged 66–75 years

**Demographics**	**Any Screening**			**Colonoscopy**			**FIT/gFOBT**			**mt-sDNA**		
	**Total incidence**	Incidence rate*	**95% CI**	**Total incidence**	Incidence rate*	**95% CI**	**Total incidence**	Incidence rate*	**95% CI**	**Total incidence**	Incidence rate*	**95% CI**
Age (y)												
66–69	74,185	94.1	(93.4,94.7)	17,038	21.6	(21.3,21.9)	44,945	57.0	(56.5,57.5)	14,092	17.9	(17.6,18.2)
70–75	65,134	94.6	(93.9,95.2)	12,979	18.8	(18.5,19.2)	39,067	56.7	(56.2,57.3)	14,869	21.6	(21.2,21.9)
Sex												
Male	52,601	76.0	(75.4,76.7)	11,169	16.1	(15.8,16.4)	32,654	47.2	(46.7,47.7)	10,043	14.5	(14.2,14.8)
Female	86,718	110.4	(109.7,111.0)	18,848	24.0	(23.6,24.3)	51,358	65.4	(64.8,65.9)	18,918	24.1	(23.7,24.4)
Race/ethnicity												
White	116,432	95.4	(94.9,95.9)	25,249	20.7	(20.4,20.9)	68,176	55.9	(55.5,56.3)	26,176	21.4	(21.2,21.7)
Black	8417	72.8	(71.3,74.3)	2120	18.3	(17.6,19.1)	5400	46.7	(45.5,47.9)	1087	9.4	(8.8,10.0)
Asian	3645	113.4	(109.9,116.9)	496	15.4	(14.1,16.8)	2908	90.5	(87.3,93.6)	300	9.3	(8.3,10.4)
Hispanic	2916	101.9	(98.4,105.4)	416	14.5	(13.2,15.9)	2429	84.9	(81.7,88.1)	116	4.1	(3.3,4.8)
Other	7909	97.8	(95.7,99.8)	1736	21.5	(20.5,22.5)	5099	63.0	(61.4,64.7)	1282	15.9	(15.0,16.7)
Regions												
Northeast	22,568	90.6	(89.5,91.7)	5310	21.3	(20.7,21.9)	13,615	54.6	(53.8,55.5)	4293	17.2	(16.7,17.7)
Midwest	29,287	89.7	(88.8,90.7)	7224	22.1	(21.6,22.6)	15,058	46.1	(45.4,46.9)	7657	23.5	(22.9,24.0)
South	54,400	95.1	(94.3,95.9)	11,458	20.0	(19.7,20.4)	31,961	55.9	(55.3,56.5)	12,596	22.0	(21.6,22.4)
West	32,542	100.9	(99.9,102.0)	5961	18.5	(18.0,19.0)	22,916	71.1	(70.2,72.0)	4404	13.7	(13.3,14.1)
Missing	522	68.1	(62.4,73.7)	64	8.3	(6.3,10.4)	462	60.2	(54.9,65.6)	11	1.4	(0.6,2.3)
Payer Type												
Medicare only	125,878	95.5	(95.0,96.0)	28,113	21.3	(21.1,21.6)	73,753	56.0	(55.6,56.4)	27,486	20.9	(20.6,21.1)
Dual Medicare & Medicaid	13,441	84.0	(82.7,85.4)	1904	11.9	(11.4,12.4)	10,259	64.1	(62.9,65.3)	1475	9.2	(8.8,9.7)

*CI* Confidence interval, *FIT/gFOBT* Fecal immunochemical test or guaiac-based fecal occult blood test, *mt-sDNA* Multitarget stool DNA test

^*^ Number of incidences per 1000 Medicare beneficiaries at average-risk

Results showing factors associated with incident screening are presented Table 3. Compared with unscreened individuals, those screened were more likely to be female and of non-Black race, and to have comorbidities such as hypertension and hyperlipidemia. The OR of incident screening for female sex was 1.48; referenced to White race, the ORs were 0.78 for Black and 1.18 for Asian; the ORs were 1.20 and 1.42 for those with hypertension and hyperlipidemia, respectively. Compared with the 2016 study sample, the ORs were 1.12 and 1.22 for the 2017 and 2018 study samples, respectively.

**Table 3:** Odds ratios of incident screening in average-risk 2016–2018 20% Medicare beneficiaries aged 66–75 years

	**Odds ratio**	**95% confidence limit**	
**Age group**			
66–69 years	1.00		
70–75 years	1.01	1.00	1.02
**Sex**			
Male	1.00		
Female	1.48	1.47	1.49
**Race/ethnicity**			
White	1.00		
Black	0.78	0.77	0.80
Asian	1.18	1.15	1.21
Hispanic	1.12	1.09	1.15
Other	1.07	1.05	1.09
**Cohort Years**			
2016	1.00		
2017	1.12	1.12	1.13
2018	1.22	1.21	1.23
**Regions**			
Northeast	1.00		
Midwest	0.98	0.97	0.99
South	1.05	1.04	1.06
West	1.12	1.10	1.13
Missing	0.76	0.72	0.81
**Payer Type**			
Medicare only	1.00		
Dual Medicare/Medicaid	0.80	0.79	0.81
**Prevalent baseline comorbidity**			
DM	0.97	0.96	0.98
ASHD	0.87	0.86	0.88
Heart failure	0.79	0.78	0.81
CVA/TIA	0.87	0.85	0.89
PVD	0.95	0.94	0.97
Cardiac other	1.09	1.07	1.10
Anemia	1.01	1.00	1.02
Hypertension	1.20	1.19	1.21
Hyperlipidemia	1.42	1.41	1.43
CKD	0.96	0.95	0.97
Cancer (nonskin)	1.08	1.07	1.10
Liver disease	1.04	1.01	1.07
COPD	0.91	0.89	0.92
GI bleeding	0.89	0.84	0.94

*ASHD* Arteriosclerotic heart disease, *CKD* Chronic kidney disease, *COPD* Chronic obstructive pulmonary disorder, *CVA/TIA* Cerebrovascular accident/transient ischemic attack, *DM* Diabetes mellitus, *GI* Gastrointestinal, *PVD* Peripheral vascular disease

### Unadjusted HCRU, Medicare costs, and baseline characteristics by screening types

Table 4 presents unadjusted PPPY HCRU and Medicare cost for the 2018 study sample by CRC screening types. Among the three CRC screening types, those who underwent a colonoscopy had the highest PPPY HCRU and Medicare costs; those who used mt-sDNA for screening had the lowest PPPY HCRU and Medicare costs. Statistical tests showed these mean differences were significant except for Medicare cost in durable medical equipment. For example, the mean number of hospital admissions in 2018 was 0.17, 0.13, and 0.11; mean physician visits were 25.83, 20.72, and 20.05; mean total Medicare Parts A and B spending in 2018 was $9140, $6631, and $6015 for individuals by colonoscopy, FIT/gFOBT, and mt-sDNA, respectively; mean total Medicare Parts A, B, and D spending in 2018 was $10,546, $8097, and $7258 for individuals by colonoscopy, FIT/gFOBT, and mt-sDNA, respectively.

**Table 4:** Healthcare resource utilization and Medicare costs in 2018 Medicare beneficiaries aged 66–75 years

	**Colonoscopy**		**FIT/gFOBT**		**mt-sDNA**	
	**Statistic**	**Variance**	**Statistic**	**Variance**	**Statistic**	**Variance**
**Medicare Parts A and B costs $PPPY**						
Total Mean, SD	9140.1	68,765.6	6630.8	18,864.2	6014.8	13,648.1
Total Median, IQR	3261.2	(1831.0, 6904.2)	1831.0	(800.5, 4742.3)	2221.4	(1236.6, 4854.9)
Inpatient						
Mean, SD	2982.1	64,987.7	2026.3	12,533.5	1481.7	7904.1
Median, IQR	0.0	(0.0,0.0)	0.0	(0.0,0.0)	0.0	(0.0,0.0)
Skilled nursing facility						
Mean, SD	247.5	2833.7	221.1	2608.6	140.0	2117.7
Median, IQR	0.0	(0.0,0.0)	0.0	(0.0,0.0)	0.0	(0.0,0.0)
Home health						
Mean, SD	174.3	1145.7	202.6	1258.7	149.3	1055.7
Median, IQR	0.0	(0.0,0.0)	0.0	(0.0,0.0)	0.0	(0.0,0.0)
Outpatient						
Mean, SD	2193.8	5515.0	1515.1	5428.3	1331.1	4279.6
Median, IQR	885.5	(189.6,2086.4)	257.7	(0.0,1029.0)	233.5	(0.0,952.8)
Any physician visit						
Mean, SD	3383.2	6402.9	2497.6	5242.3	2763.3	5623.7
Median, IQR	1996.9	(1103.0,3719.6)	1225.4	(529.9,2786.8)	1655.3	(1015.5,3094.2)
Durable medical equipment						
Mean, SD	159.3	1194.0	168.3	1836.5	149.4	1067.8
Median, IQR	0.0	(0.0,0.0)	0.0	(0.0,0.0)	0.0	(0.0,0.0)
**All-cause HCRU, count PPPY**						
Hospitalization						
Mean, SD	0.17	0.56	0.13	0.48	0.11	0.41
Median, IQR	0.00	(0.00,0.00)	0.00	(0.00,0.00)	0.00	(0.00,0.00)
Total hospital days, all patients						
Mean, SD	0.86	4.73	0.62	3.82	0.46	2.62
Median, IQR	0.00	(0.00,0.00)	0.00	(0.00,0.00)	0.00	(0.00,0.00)
Total hospital days, hospitalized patients						
Mean, SD	7.28	11.20	6.68	10.79	5.51	7.35
Median, IQR	4.00	(2.00,8.00)	3.00	(2.00,7.00)	3.00	(2.00,6.00)
OP, ED/Observational stay						
Mean, SD	0.30	0.79	0.26	0.74	0.22	0.60
Median, IQR	0.00	(0.00,0.00)	0.00	(0.00,0.00)	0.00	(0.00,0.00)
OP, non-ED/Observational stay						
Mean, SD	4.86	5.94	3.80	5.58	3.43	4.93
Median, IQR	3.00	(1.00,6.00)	2.00	(0.00,5.00)	2.00	(0.00,4.00)
Any physician visit						
Mean, SD	25.83	24.46	20.72	22.47	20.05	19.43
Median, IQR	19.00	(11.00,32.00)	14.00	(8.00,26.00)	14.00	(8.00,25.00)

*ED* Emergency department, *FIT/gFOBT* Fecal immunochemical test or guaiac-based fecal occult blood test, *HCRU* Healthcare resource utilization, *IQR* Interquartile range, *mt-sDNA* Multitarget stool DNA test, *OP* Outpatient, *PPPY* Per person per year, *SD* Standard deviation

Except for mean Medicare cost in durable medical equipment, other cost categories and utilizations were significantly different with all *P* values < .01

Additional file 1: Supplementary Table 2, presents demographic and baseline characteristics for the 2018 study sample by CRC screening types. Among the three screening types, individuals who underwent mt-sDNA had higher median age, higher percentages of female sex or White race, and higher percentage living in the Midwest and South. Those who underwent FIT/gFOBT had higher prevalence of chronic conditions of diabetes, hypertension, hyperlipidemia, and chronic kidney disease; prevalence of other baseline conditions was similar by different screening types.

## Discussion

Although prevalence of up-to-date screening status is the usual reported statistic, annual incident screening may better reflect current clinical practices and may be more actionable. For example, annual incident screening does not reflect colonoscopies performed up to 9 years prior to the year being measured. Similarly, interventions to improve CRC screening rates can only affect future care; the magnitude of any resulting change is difficult to detect when up to 9 years of prior screening utilization are included in the outcome measure. To our knowledge, this is the first study to report the incident CRC screening rates among the older average-risk population. In two previous studies, researchers reported that among newly insured Medicaid members in Oregon turning 50 years old, the incidence of any type of screening was 17% within 1 year and 35% within 4 years [10, 11]. The value of this metric to facilitate quality assessment and improvement requires additional study for confirmation. In our study using the most current Medicare 20% random sample data, we found that the overall age-, sex-, and race-adjusted incident CRC screening rate per 1000 Medicare average-risk beneficiaries aged 66–75 years increased from 85.2 in 2016 to 94.3 in 2018. This overall increase might be driven by the increasing use of mt-sDNA. From 2016 to 2018, the incident screening rate by mt-sDNA increased 201.5%; the rate by colonoscopy and FIT/gFOBT decreased 11.4% and 2.4%, respectively.

We also found sex and race/ethnicity differences in incident CRC screening overall and by screening type. For overall screening, women were about 50% more likely to be screened than men. For overall incident screening by race/ethnicity, Asian individuals had the highest screening rate followed by Hispanic individuals; Black individuals were the least likely to be screened among race/ethnicity groups even after adjusting for demographic and clinical factors. By screening type, we found different screening patterns among race/ethnicity groups. For colonoscopy, White individuals had the highest incident screening rate, which was 1.13, 1.34, and 1.42 times the rates of Black, Asian, and Hispanic individuals, respectively. For FIT/gFOBT, Asian individuals had the highest screening rate, which was 1.62 and 1.94 times the rates of White and Black individuals, respectively (and slightly higher than that of Hispanic individuals). For mt-sDNA, White individuals had the highest incident screening rate, which was 2.28, 2.30, and 5.29 times the rates of Black, Asian, and Hispanic individuals, respectively. This different pattern in use of CRC screening type might reflect different preferences or accessibilities among different race/ethnicity groups.

Unadjusted results on overall HCRU and Medicare costs showed differences among average-risk individuals who underwent different CRC screening types. For Medicare beneficiaries who had a colonoscopy in 2018, their mean Medicare Parts A and B spending was $9140, which was about $2509 and $3125 more than those who had a FIT/gFOBT and mt-sDNA, respectively. Higher spending in those who underwent a colonoscopy might mainly be due to some additional procedures associated with the screening (eg, finding and removing a polyp). For those who had FIT/gFOBT performed in 2018, the total and different types of utilizations and mean spending were slightly higher compared with those who had mt-sDNA—except that mean (median) annual spending on physician visits among those who had FIT/gFOBT was $251 ($406) lower compared with those who had mt-sDNA. These differences might not be explained by an individual’s health profile because prevalence of baseline comorbidities was almost the same, except that those who used FIT/gFOBT had a slightly higher prevalence of diabetes, hypertension, and hyperlipidemia.

Our study has some limitations. First, due to lack of Medicare data from the early years, we could not examine trends of incident screening rate over time; in particular, we could not show if the pattern changed among different race/ethnicity groups. Second, as an observational study, residual confounding may have occurred and causality cannot be assessed. Third, the differences in HCRU and costs by different screening types could not be explained as cost-effectiveness by screening types and should be interpreted very carefully. Fourth, when we examined whether a beneficiary had a colonoscopy in the prior 9 years, we might not have had complete Medicare claims data for the beneficiaries aged younger than 74 years with the original reason for Medicare enrollment as aged. However, this may not be a big concern because, under the current guideline and insurance coverage policy, a person who had a colonoscopy for screening would not have another colonoscopy for the purpose of screening during the next 10 years. Last, these results may not be generalizable to the Medicare Advantage population.

Findings from this study have important public health implications. The overall observed incident CRC screening rate in average-risk Medicare beneficiaries in 2016–2018 was low although increasing. There is a big gap between current screening and the USPSTF recommendations. For example, the 2018 total number of Medicare beneficiaries aged 65–74 years and enrolled in the original FFS with Part A or B coverage was 18,967,577 [15]. Based on our study sample selection result, we estimated there were approximately 10.54 million average-risk beneficiaries who qualified for incident screening in 2018. However, only about 1 million of these had CRC screening (10.54 million multiplied by 94.3 per 1000) in this age group; more than 9.5 million beneficiaries did not undergo timely screening. If we consider both original FFS and Medicare Advantage, the 2018 total enrollment for age 65–74 years was 29,063,456 [15]. Similarly, we estimated that there were more than 14.6 million average-risk beneficiaries aged 65–74 years who did not undergo timely screening in 2018. In Healthy People 2030, the US government targets 74.4% of adults for CRC screening, based on the up-to-date rate of 65.2% in 2018 [16, 17]. Our study results provide evidence of gaps between real-world CRC screening and the governmental target, although these two measures are different.

## Conclusions

The observed incident CRC screening rate in average-risk Medicare beneficiaries aged 66–75 years in 2016–2018, while increasing, was still low. Our findings suggest more work is needed to improve CRC screening overall and, especially, among male and Black Medicare beneficiaries.

## Supplementary Information

**Additional file 1: Supplementary Appendix 1.** Codes for excluding high-risk diseases for colorectal cancer (CRC) screening. **Supplementary Appendix 2.** Codes for colorectal cancer (CRC) screenings and tests. **Supplementary Appendix 3.** Codes for comorbid conditions. **Supplementary Table 1.** Attrition table for 2016-2018 sample selections. **Supplementary Table 2.** Demographic characteristics and comorbid conditions by screening types for 2018 Medicare beneficiaries aged 66-75 years at average risk.

## Data Availability

All data that support the findings of this study are included within the article (and any supplementary files). Because analytic data files for this manuscript include restricted Medicare data, they are subject to data use agreements with the Centers for Medicare & Medicaid Services (CMS) and, as such, not available for distribution.

## Abbreviations

CI: Confidence interval
CPT: Current Procedural Terminology
CRC: Colorectal cancer
ED: Emergency department
FIT/gFOBT: Fecal immunochemical test or guaiac-based fecal occult blood test
FFS: Fee-for-service
HCPCS: Healthcare Common Procedure Coding System
HCRU: Healthcare resource utilization
ICD-9/10-CM: International Classification of Diseases, Ninth/Tenth Revision, Clinical Modification
mt-sDNA: Multitarget stool DNA
OR: Odds ratio
PPPY: Per person per year
USPSTF: US Preventive Services Task Force
