# Supplementary material for: Incident colorectal cancer screening and associated healthcare resource utilization and Medicare cost among Medicare beneficiaries aged 66–75 years in 2016–2018
Source: BMC Health Serv Res. 2022 Oct 3;22:1228. doi: 10.1186/s12913-022-08617-8 (PMC9531423; doi:10.1186/s12913-022-08617-8)
Supplement: Supplementary file 1 — Additional file 1: Supplementary Appendix 1. Codes for excluding high-risk diseases for colorectal cancer (CRC) screening. Supplementary Appendix 2. Codes for colorectal cancer (CRC) screenings and tests. Supplementary Appendix 3. Codes for comorbid conditions. Supplementary Table 1. Attrition table for 2016-2018 sample selections. Supplementary Table 2. Demographic characteristics and comorbid conditions by screening types for 2018 Medicare beneficiaries aged 66-75 years at average risk. [file 12913_2022_8617_MOESM1_ESM.docx]

Li S, Miller-Wilson L-A, Guo H, Hoover M, Fisher DA. Incident colorectal cancer screening and associated healthcare resource utilization and Medicare cost among Medicare beneficiaries aged 66-75 years in 2016-2018.

**Additional File 1**

**Supplementary Appendix 1. Codes for excluding high-risk diseases for colorectal cancer (CRC) screening**

| **Exclusion Categories** | **Subcategory** | ***ICD-9-CM* diagnosis codes, *ICD-10-CM* diagnosis codes, and *CPT*/HCPCS codes** |
| --- | --- | --- |
| **High-risk diagnosis** | Colorectal polyp | *ICD-9-CM* diagnosis codes: 2113, 2114, 5690, 5564, 56949, 20950, 20952, 20951, 20953, 20954, 20955, 20956, 20957  *ICD-10-CM* diagnosis codes: D120, D121, D122, D123, D124, D125, D126, D127, D128, K5140, K51411, K51412, K51413, K51414, K51418, K51419, K635, K620, K621, D3A029, D3A020, D3A021, D3A022, D3A023, D3A024, D3A025, D3A026 |
|  | Colorectal polyp - history | *ICD-9-CM* diagnosis codes: V1272  *ICD-10-CM* diagnosis codes: Z86010 |
|  | Irritable bowel disease | *ICD-9-CM* diagnosis codes: 5550, 5551, 5552, 5559, 5560, 5561, 5562, 5563, 5565,  5566, 5568, 5569  *ICD-10-CM* diagnosis codes: K5000, K50011, K50012, K50013, K50014, K50018, K50019, K5010, K5011, K50111, K50112, K50113, K50114, K50118, K50119, K508, K5080, K50811, K50812, K50813, K50814, K50818, K50819, K5090, K50911, K50912, K50913, K50914, K50918, K50919, K5100, K51011, K51012, K51013, K51014, K51018, K51019, K5120, K51211, K51212, K51213, K51214, K51218, K51219, K513, K5130, K51311, K51312, K51313, K51314, K51318, K51319, K5150, K51511, K51512, K51513, K51514, K51518, K51519, K5180, K51811, K51812, K51813, K51814, K51818, K51819, K5190, K51911, K51912, K51913, K51914, K51918, K51919, K5289 |
|  | CRC | *ICD-9-CM* diagnosis codes: 1530, 1531, 1532, 1533, 1534, 1535, 1536, 1537, 1538, 1539, 1540, 1541, 1548, 1975, 2303, 2304, 20910, 20911, 20912, 20913, 20914, 20915, 20916, 20917  *ICD-10-CM* diagnosis codes: C180, C181, C182, C183, C184, C185, C186, C187, C188, C189, C19, C20, C212, C218, C785, C7A020, C7A021, C7A022, C7A023, C7A024, C7A025, C7A026, C7A029, D0140, D0149, D010, D011, D012 |
|  | History of CRC | *ICD-9-CM* diagnosis codes: V1005, V1006  *ICD-10-CM* diagnosis codes: Z85030, Z85038, Z85040, Z85048, Z8509 |
|  | Family history of GI cancer | *ICD-9-CM* diagnosis code: V160  *ICD-10-CM* diagnosis code: Z800 |
|  | Other HR evidence | *ICD-9-CM* diagnosis code: V8409  *ICD-10-CM* diagnosis code: Z1509 |
| **High-risk symptom** |  | *ICD-9-CM* diagnosis codes: 5781, 7921  *ICD-10-CM* diagnosis codes: K625, K921, R195 |
| **CRC procedure for exclusion** | Colonoscopy | *CPT*/HCPCS: 44388, 44389, 44390, 44391, 44392, 44393, 44394, 44397, 44401, 44402, 44403, 44404, 44405, 44406, 44407, 44408, 45379, 45382, 45386, 45387, 45389, 45393, 45398, G6025, G0105, G6019, G6020 |
|  | Sigmoidoscopy | *CPT*/HCPCS: 45332, 45334, 45337, 45340, 45345, 45347, 45350, 45303, 45307, 45317, 45321, 45327, G6023 |
|  | Virtual colonoscopy (colonography)/ CT scan of colon | *CPT*/HCPCS: 74261, 74262, 0067T |
|  | Barium enema | *CPT*/HCPCS: G0120 |

**Supplementary Appendix 2. Codes for colorectal cancer (CRC) screenings and tests**

| **Test Type** | ***CPT* or HCPCS Codes** |
| --- | --- |
| Colonoscopy | 45355, 45378, 45391, G0121, 45380, 45381, 45383, 45384, 45385, 45388, 45390, 45392, G6024 |
| Sigmoidoscopy | 45330, 45341, 45300, G0104, 45331, 45333, 45335, 45338, 45339, 45342, 45346, 45349, 45305, 45308, 45309, 45315, 45320, G6022 |
| FIT | 82274, G0328 |
| gFOBT | 82270, G0107 |
| mt-sDNA | 81528, G0464, S3890 |

**Supplementary Appendix 3. Codes for comorbid conditions**

| **Comorbid condition** | ***ICD-9-CM* diagnosis codes** | ***ICD-10-CM* diagnosis codes** |
| --- | --- | --- |
| Diabetes | 250; 3572; 3620x; 36641 | E08.31-E08.36; E08.40; E08.42; E09.31-E09.36; E09.40; E09.42; E10; E11; E13 |
| Arteriosclerotic heart disease | 410-414; V45.81; V45.82 | I12-I22; I24-I25; Z95.1; Z95.5; Z98.61 |
| Heart failure | 398.91;422; 425; 428; 402.X1; 404.x1; 404.x3; V42.1 | I09.81; I11.0; I13.0; I40-I43; I50; Z48.21; Z48.280, Z94.1, Z94.3 |
| Cerebrovascular accidents/transient ischemic attack | 430-438 | G45; F46; I60- I63; I65- I66; I67.1-I67.2; I67.4-I67.7; I67.81; I67.82; I67.841; I67.848; I67.89; I67.9; I68-I69 |
| Peripheral vascular disease | 440-444; 447; 451-453; 557 | E08.51; E08.52; E09.51; E09.52; E10.51; E10.52; E11.51; E11.52; E13.51; E13.52; I67.0; I70-I74; I77; I79; I80.0; I80.1; I80.20-I80.23; I80.29; I80.3; I80.8; I80.9; I81; I82.0-I82.3; I82.40-I82.44; I82.49; I82.4Y; I82.4Z; I82.50-I82.54; I82.59; I82.5Y; I82.5Z; I82.6-I82.9; I82.A; I82.B; I82.C; K55.0; K55.1; K55.3; K55.8; K55.9; M31.8; M31.9 |
| Cardiac other | 420-421;423-424; 429; 785.0-785.3; V42.2; V43.3 | A18.84; I23; I25.10; I30-I39; I51- I52; I97.0-I97.1; M32.11; M32.12; R00.0; R00.2; R00.8; R00.9; R01.0; R01.1; R01.2; Z95.2-Z95.4 |
| Anemia | 280-285 | D50-D53; D55-D56; D57.00-D57.02; D57.1; D57.20; D57.211; D57.212; D57.219; D57.3; D57.40; D57.411; D57.412; D57.419; D57.80; D57.811; D57.812; D57.819; D58-D64 |
| Hypertension | 362.11; 401-405; 437.2 | H35.03; I10-I13;, I15 -I16, I67.4, N26.2 |
| Hyperlipidemia | 272 | E71.30; E75.21; E75.22; E75.24; E75.3; E75.5; E75.6; E77; E78.0-E78.6; E78.70; E78.79; E78.8; E78.9; E88.1; E88.2; E88.89 |
| Kidney disease | 016.0; 095.4; 189.0,189.9; 223.0; 236.91; 250.4; 271.4; 274.1; 283.11; 403; 404; 440.1; 442.1; 447.3; 572.4; 580-588; 591; 642.1; 646.2; 753.12-753.19; 753.2; 794.4 | A18.11; A52.75; B52.0; C64; C68.9; D30.0; D41.0-D41.2; D59.3, E08.2; E09.2; E10.2; E10.65; E11.2; E11.65; E13.2; E74.8; I12; I13; I70.1; I72.2; I77.3; K76.7; M10.3; M32.14; M32.15; M35.04; N00-N08; N13.0-N13.2; N13.30; N13.39; N14-N19; N20.0; N25; N26; O10.4; O12.1; O12.2; O26.83; O90.89; Q61.02; Q61.1-Q61.5, Q61.8; Q62.0-Q62.3; R94.4 |
| Cancer, excluding nonmelanoma skin cancer | 140-165; 170-172; 174-176 except 176.0; 179-198—209 except 209.3x and 209.75; 230-231; 233-238 except 238.2 | C00-C26; C30-C34; C37-C41; C43; C45-C45; C46.1-C46.5; C46.7; C46.9; C47-C58; C60-C86; C88; C90-C96; D00-D03; D05-D08; D09 |
| Liver disease | 570; 571; 572.1; 572.4; 573.1-573.3; V42.7 | B25.1; K70-K71; K72.0; K73-K77; Z48.23; Z94.4 |
| Chronic obstructive pulmonary disease | 491-494; 496; 510 | J41-J45; J47; J86 |
| Gastrointestinal bleeding | 456.0-456.2; 530.7; 531-534; 569.84-569.85; 578; 537.83; 537.84; 535.x1; 530.21; 530.82 | I85; K22.11; K22.6; K22.8; K25-K28; K29.01; K29.21; K29.31; K29.41; K29.51; K29.61; K29.71; K29.81; K29.91; K31.811; K31.82; K52.81; K55.20; K55.21; K56.699; K92.0-K92.2 |

**Supplementary Table 1. Attrition table for 2016-2018 sample selections**

| **Sample selection with inclusion/exclusion criteria** | **Number of subjects after each inclusion/exclusion criteria applied** | | | **Percent of subjects remained after inclusion/exclusion criteria applied** | | |
| --- | --- | --- | --- | --- | --- | --- |
|  | **2016** | **2017** | **2018** | **2016** | **2017** | **2018** |
| Having Medicare Parts A & B coverage and alive on Jan. 1 of current year (index year) | 6,475,346 | 6,573,911 | 6,576,062 | 100.0 | 100.0 | 100.0 |
| Continuously enrolled in Medicare Part A & B in previous year (baseline year) | 5,935,918 | 6,047,844 | 6,051,732 | 91.7 | 92.0 | 92.0 |
| No HR diagnosis codes during baseline year | 5,322,727 | 5,374,393 | 5,356,837 | 82.2 | 81.8 | 81.5 |
| No HR symptom diagnosis codes during baseline year | 5,223,752 | 5,234,300 | 5,213,383 | 80.7 | 79.6 | 79.3 |
| No CRC procedure codes during baseline year | 5,219,631 | 5,230,097 | 5,209,280 | 80.6 | 79.6 | 79.2 |
| No hospice claims during index year | 5,082,904 | 5,091,532 | 5,070,736 | 78.5 | 77.5 | 77.1 |
| No colonoscopy performed within 9 years before index year ^a^ | 3,291,282 | 3,356,375 | 3,357,497 | 50.8 | 51.1 | 51.1 |
| No FS performed within 4 years before index year ^a^ | 3,275,389 | 3,341,566 | 3,343,068 | 50.6 | 50.8 | 50.8 |
| No mt-sDNA test within 2 years before index year ^a^ | 3,268,959 | 3,320,961 | 3,300,222 | 50.5 | 50.5 | 50.2 |
| No CRC test on or after a HR/CRC event during index year ^a^ | 3,103,043 | 3,152,667 | 3,132,956 | 47.9 | 48.0 | 47.6 |
| **Aged 66-75 years as of Jan. 1 of index year - final study sample** | **1,424,453** | **1,468,177** | **1,477,638** | **22.0** | **22.3** | **22.5** |

CRC, colorectal cancer; FS, flexible sigmoidoscopy; HR, high risk; mt-sDNA, multitarget stool DNA test

^a^ Medicare claims were used to determine screenings in prior years.

**Supplementary Table 2. Demographic characteristics and comorbid conditions by screening types for 2018 Medicare beneficiaries aged 66-75 years at average risk**

|  | **Colonoscopy** | **FIT/gFOBT** | **mt-sDNA** |
| --- | --- | --- | --- |
| **Total patients** | 30,017 (100.00) | 84,012 (100.00) | 28,961 (100.00) |
| **Age (in years)** |  |  |  |
| Mean (SD) | 69.39 (2.64) | 69.58 (2.72) | 69.84 (2.69) |
| Median (IQR) | 69.00 (67.00,71.00) | 69.00 (67.00,72.00) | 70.00 (68.00,72.00) |
| **Sex** |  |  |  |
| Male | 11,169 (37.21) | 32,654 (38.87) | 10,043 (34.68) |
| Female | 18,848 (62.79) | 51,358 (61.13) | 18,918 (65.32) |
| **Race/ethnicity** |  |  |  |
| White | 25,249 (84.12) | 68,176 (81.15) | 26,176 (90.38) |
| Black | 2120 (7.06) | 5400 (6.43) | 1087 (3.75) |
| Asian | 496 (1.65) | 2908 (3.46) | 300 (1.04) |
| Hispanic | 416 (1.39) | 2429 (2.89) | 116 (0.40) |
| Other | 1736 (5.78) | 5099 (6.07) | 1282 (4.43) |
| **Regions** |  |  |  |
| Northeast | 5310 (17.69) | 13,615 (16.21) | 4293 (14.82) |
| Midwest | 7224 (24.07) | 15,058 (17.92) | 7657 (26.44) |
| South | 11,458 (38.17) | 31,961 (38.04) | 12,596 (43.49) |
| West | 5961 (19.86) | 22,916 (27.28) | 4404 (15.21) |
| Missing | 64 (0.21) | 462 (0.55) | 11 (0.04) |
| **Payer Type** |  |  |  |
| Medicare only | 28,113 (93.66) | 73,753 (87.79) | 27,486 (94.91) |
| Dual Medicare/Medicaid | 1904 (6.34) | 10,259 (12.21) | 1475 (5.09) |
| **Charlson Comorbidity Index** |  |  |  |
| Mean (SD) | 0.61 (1.26) | 0.61 (1.23) | 0.55 (1.14) |
| Median (IQR) | 0.00 (0.00,1.00) | 0.00 (0.00,1.00) | 0.00 (0.00,1.00) |
| **Comorbid Conditions** |  |  |  |
| Diabetes mellitus | 5858 (19.52) | 19,186 (22.84) | 5730 (19.79) |
| ASHD | 3156 (10.51) | 8805 (10.48) | 3003 (10.37) |
| Heart failure | 985 (3.28) | 3418 (4.07) | 1044 (3.60) |
| CVA/TIA | 1020 (3.40) | 3234 (3.85) | 1079 (3.73) |
| PVD | 1738 (5.79) | 5387 (6.41) | 1672 (5.77) |
| Cardiac other | 4183 (13.94) | 11,849 (14.10) | 3983 (13.75) |
| Anemia | 2311 (7.70) | 6184 (7.36) | 1624 (5.61) |
| Hypertension | 14,358 (47.83) | 43,155 (51.37) | 13,912 (48.04) |
| Hyperlipidemia | 13,027 (43.40) | 38,743 (46.12) | 12,492 (43.13) |
| CKD | 3480 (11.59) | 11,119 (13.24) | 3281 (11.33) |
| Cancer ^a^ | 2283 (7.61) | 5158 (6.14) | 1779 (6.14) |
| Liver disease | 569 (1.90) | 1172 (1.40) | 303 (1.05) |
| COPD | 2457 (8.19) | 7957 (9.47) | 2697 (9.31) |
| GI bleeding | 145 (0.48) | 300 (0.36) | 51 (0.18) |

ASHD, arteriosclerotic heart disease; CKD, chronic kidney disease; COPD, chronic obstructive pulmonary disease; CVA/TIA, cerebrovascular accident or transient ischemic attack; FIT/gFOBT, fecal immunochemical test or guaiac-based fecal occult blood test; GI, gastrointestinal; IQR, interquartile range; mt-sDNA, multitarget stool DNA test; PVD, peripheral vascular disease; SD, standard deviation

^a^ Cancer excluding nonmelanoma skin cancer
